# Effectiveness of early heparin therapy on outcomes in critically ill patients with sepsis-induced coagulopathy

**DOI:** 10.3389/fphar.2023.1173893

**Published:** 2023-05-15

**Authors:** Jia-Jia Huang, Zhi-Ye Zou, Zhi-Peng Zhou, Yan Liu, Zhen-Jia Yang, Jing-Jing Zhang, Ying-Yi Luan, Yong-Ming Yao, Ming Wu

**Affiliations:** ^1^ Department of Infection and Critical Care Medicine, Health Science Center, Shenzhen Second People’s Hospital and First Affiliated Hospital of Shenzhen University, Shenzhen, China; ^2^ Postgraduate Education, Shantou University Medical College, Shantou, China; ^3^ Department of Critical Care Medicine, Pingshan District People’s Hospital of Shenzhen, Shenzhen, China; ^4^ Department of Central Laboratory, Beijing Obstetrics and Gynecology Hospital, Capital Medical University, Beijing, China; ^5^ Trauma Research Center, Medical Innovation Research Department and Fourth Medical Center of the Chinese PLA General Hospital, Beijing, China; ^6^ Department of Nosocomial Infection Prevention and Control, Shenzhen Second People's Hospital, Shenzhen, China

**Keywords:** heparin, sepsis-induced coagulopathy, disseminated intravascular coagulation, outcome, mortality

## Abstract

**Background:** This study aimed to investigate whether early unfractionated heparin (UFH) administration provides a survival advantage for patients with sepsis-induced coagulopathy (SIC).

**Methods:** Patients hospitalized with sepsis-induced coagulopathy from the Medical Information Mart for Intensive Care (MIMIC)-IV database were identified. Patients were divided into two groups, who received unfractionated heparin (UFH) subcutaneously within 24 h after intensive care unit (ICU) admission, and the control group, who received not. The primary endpoint was intensive care unit mortality, the secondary outcomes were 7, 14, and 28-day and hospital mortality. Propensity score matching (PSM) the marginal structural Cox model (MSCM) and E-value analysis were used to account for baseline differences, time-varying and unmeasured confounding factors.

**Results:** A total of 3,377 patients with sepsis-induced coagulopathy were enrolled in the study, of which 815 in unfractionated heparin group and 2,562 in control group. There was significant effect on primary and secondary outcomes with unfractionated heparin after propensity score matching (intensive care unit mortality, hazard ratio [HR] 0.69, 95% confidence interval [CI] 0.52–0.92; 7-day, HR 0.70, 95% CI 0.49–0.99; 14-day, HR 0.68.95% CI 0.50–0.92; 28-day, HR 0.72, 95% CI 0.54–0.96; hospital mortality, HR 0.74, 95% CI 0.57–0.96), marginal structural Cox model manifested unfractionated heparin associated with decreased intensive care unit mortality in all populations (HR 0.64, 95% CI 0.49–0.84), and stratification with the marginal structural Cox model indicated analysis further indicated the survival advantage only among patients with an sepsis-induced coagulopathy score of 4 (HR 0.56, 95% CI 0.38–0.81). Further analysis showed that treatment with 6,250–13750 IU/day of unfractionated heparin associated with a decreased risk of intensive care unit mortality. Similar results were replicated in subgroup analysis with propensity score matching only for patients with an sepsis-induced coagulopathy score of 4 (intensive care unit mortality, HR 0.51, 95% CI 0.34–0.76).

**Conclusion:** This study found early unfractionated heparin therapy to patients with sepsis-induced coagulopathy appears to be associated with improved outcomes. Subgroup analysis further demonstrates heparin therapy decreased intensive care unit mortality primarily in patients only with SIC score of 4.

## Introduction

Nearly 50 million patients suffer from sepsis worldwide each year, and sepsis-associated mortality (more than 11 million cases) was higher than mortality associated with ischemic heart disease (9 million cases) or tumors (10 million cases) in 2019 (Rudd et al., 2020). Sepsis mortality increases significantly when combined with coagulopathy, which represents a mounting clinical challenge for healthcare professionals. Previous studies have shown that the incidence of disseminated intravascular coagulation (DIC) is as high as 35% in patients with severe sepsis (Adamik et al., 2017). Sepsis-induced coagulopathy (SIC) is regarded as an early phase of DIC because it includes most cases of overt DIC (Iba et al., 2020), which provides the possibility for early clinical intervention of sepsis.

Unfractionated heparin (UFH) exerts anti-coagulation, anti-inflammatory, anti-complement activity, and protease regulation (Li et al., 2014). Therefore, unfractionated heparin has been widely used in clinical practice Systematic reviews have documented that treatment with a low dose of heparin is associated with a significant reduction 28-day mortality among patients with sepsis (Wang et al., 2014; Zarychanski et al., 2015). Our previous results study manifested an association between heparin administrationand decreased risk-adjusted mortality in septic patients (HR 0.70, 95% CI 0.56–0.87, *p* < 0.001) (Zou et al., 2022). Unfortunately, in update surviving sepsis campaign 2021, there were no recommendations on anticoagulation in patients with sepsis, only recommend using low molecular weight heparin over unfractionated heparin for venous thromboembolism prophylaxis (Evans et al., 2021). Therefore, the indications, timing, and dosage of unfractionated heparin administration in patients with sepsis are still unclear.

Recently, a study suggested a novel role for UFH to prevent septic coagulation and lethality by inhibiting the caspase-11 pathway (Tang et al., 2021), which has not been proven clinically. Since SIC is an early stage of septic DIC, whether anticoagulation treatment would benefit patients with SIC remains largely unknown. Therefore, we evaluate the effectiveness and dosage of UFH in patients with SIC using data from MIMIC-IV.

## Materials and methods

### Data source and study design

We retrospectively collected data following the MIMIC-IV (version 2.0), which included two in-hospital database systems: the custom whole-hospital electronic health record (EHR) and ICU-specific clinical information. These systems contain the integrative de-identified patient clinical information admitted into ICUs in Beth Israel. Deaconess Medical Center (Boston, Massachusetts) during 2008–2019. Database access was granted to a candidate who passed the collaborative institutional training initiative examination (certification number 38995627 for author Huang).

### Participants

From 2008 to 2019, 315,460 individuals were admitted to ICUs. The patient eligibility criteria were as follows: 1) age ≥18 years; 2) sepsis according to Sepsis 3.0 criteria, i.e., a suspicious infection plus a sharply elevated Sequential. Organ Failure Assessment (SOFA) score ≥2 (Singer et al., 2016), and 3) had a SIC score ≥4 (Supplementary Table S1) within the first 24-h (h) following admission into ICU.

Patient exclusion criteria included the following: 1) age <18 years; 2) ICU stay <24 h; 3) multiple ICU admissions; 4) thrombotic diseases, high risk of thrombosis; 5) pregnancy; 6) unfractionated heparin application in other usage such as dialysis, or low molecular weight heparin (LMWH) administration or warfarin treatment during the ICU stay; 7) exposed to unfractionated heparin in the other time but not the first 24 h.

### Research strategy and definitions

Using Structured Query Language, the MIMIC-IV database was populated with data. The database was searched using techniques described previously, and the extracted patient data were then analyzed (Shen et al., 2019; Zou et al., 2022). Patients with multiple hospitalizations were only counted for their initial hospitalization. On day one of ICU admission, age at admission, gender, weight, ethnicity, laboratory results (white blood cell [WBC] count, platelet count, hemoglobin, international normalized ratio [INR], partial thromboplastin time [PTT]), vital signs (heart rate, mean arterial pressure [MAP], respiratory rate and temperature), and comorbidities (diabetes mellitus (DM), hypertension, chronic pulmonary disease (CPD), and chronic heart disease [CHD]), vasopressor use, mechanical ventilation, SIC score, lengths of hospital and ICU stay. Clinical severity scales, such as the SOFA score and Simplified Acute Physiology Score II(SAPS II), were collected. Notably, we determined the SOFA score within 24 h of ICU admission.

Laboratory variables APTT was analyzed throughout the ICU stay. The database was utilized to extract chart times and physiological levels for measurements. If cases were measured multiple times, the maximum daily INR value and the minimum daily platelet count were selected for analysis. None of these screening variables had a rate of missing data exceeding 10% (Supplementary Table S2). Variables with less than 10% of missing data were subject to single imputation.

### Exposure and endpoints

Cases were divided into two groups: unfractionated heparin (cases receiving subcutaneous unfractionated heparin 24 h after ICU administration) and control (cases not receiving unfractionated heparin during ICU stays). Our primary endpoint was ICU morality, with 7-day, 14-day, 28-day, and hospital mortality serving as secondary outcomes.

### Statistical analysis

Analyzing categorical data represented as numbers or percentages using Fisher’s exact or chi-square tests between two groups. Continuous data were portrayed using mean (standard deviation, SD) or median (interquartile range [IQR]).

We utilized propensity score matching (PSM) to examine fundamental differences in the probability of receiving UFH. The PSM measures the probability of a patient receiving UFH therapy. The UFH group in the PSM received UFH 24 h after ICU admission. Using nearest-neighbor matching, treated cases were compared to untreated cases. Before and after matching, the standardized mean difference (SMD) was calculated to determine if PSM reduced pre-treatment covariate differences between groups (Ostermann et al., 2020). A Cox proportional hazards model was adopted to adjust residual imbalance by incorporating factors satisfying *p* < 0.05 and potential clinical.expertise-judged confounding.

UFH administration upon ICU admission was identified as a time-dependent variable in the marginal structural Cox model (MSCM). Possible basic confounders, such as age, sex, mechanical ventilation (MV), vasopressor use, SOFA, and SAPS II scores, were assessed 24 h after ICU admission. APTT were considered time-dependent confounders throughout the ICU admission and incorporated into this model. MSCM parameters were predicted using inverse probability weighting (IPW) to correct for confounding in addition to types of selection bias, such as informative censoring (Shinozaki and Suzuki, 2020). IPW was performed to weigh each case, which allowed for the creation of two pseudopulations that were close to time-dependent and fundamental confounders and unfractionated heparin administration differences. Electronic Supplementary Material (ESM) displays IPW and R code information in MSCM alongside R code information. IPW package was used to estimate inverse probability weights (Grafféo et al., 2019).

We also performed stratification analyses to determine whether UFH use and ICU mortality varied by gender, age, ethnicity, ventilation and SIC subgroup classification. The Cox model (after adjusting for each patient’s basic variable) was utilized for subgroup analysis. E-values were computed to assess the likelihood of unmeasured confounding between UFH and ICU mortality (Haneuse et al., 2019). E-values quantify the magnitude required to negate the association between unfractionated heparin and ICU mortality due to one unmeasured confounder. MSCM was used to perform a number of subgroup analyses that were predefined, and Two-sided *p* < 0.05 signified statistical significance. The R package (4.1.1) was used for statistical analysis.

## Results

### Characteristics of patients on the baseline

**FIGURE 1:**
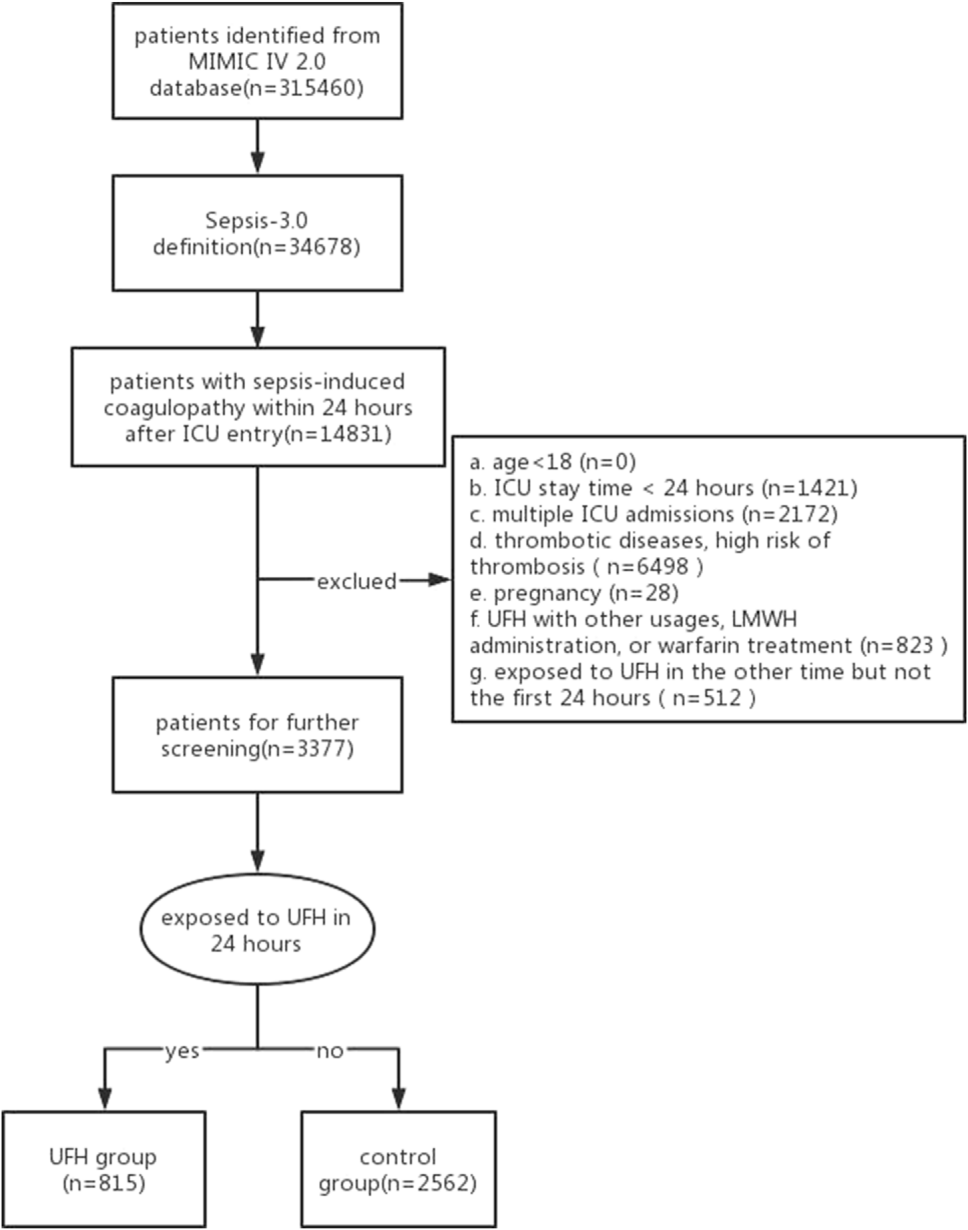
Flowchart of patient selection.

The original search yielded 315,460 ICU admissions based on the MIMIC-IV 2.0 database. In total, 34,678 cases were diagnosed with sepsis, while 3,377 were diagnosed with SIC within 24 h of ICU admission. In our cases, 815 patients received heparin within the first 24 h after ICU admission, while 2,562 did not received (Figure 1).

Except for weight, age, ethnicity, chronic heart disease, MAP, APTT and WBC, the other variables in Table 1 revealed significant differences between the two groups. Notably, there were significantly more critically ill patients in the unfractionated heparin group than in the control. group (SOFA score of 8 (Zarychanski et al., 2015; Singer et al., 2016) vs. 6 (Zarychanski et al., 2015; Tang et al., 2021), *p* < 0.001). Non-heparin cases may be more likely to utilize vasopressors (65.8% vs. 52.9%; *p* < 0.001) and require MV (49.8% vs. 40.9%, *p* < 0.001).

**TABLE 1:** Baseline characteristics of patients with sepsis-induced coagulopathy before and after propensity score matching.

		Propensity score matching							
		Before				After			
Characteristics of patients	Overall(n = 3,377)	Control group (n = 2,562)	UFH group (n = 815)	*p*-value	SMD	Control group (n = 784)	UFH group (n = 784)	*p*-value	SMD
Demographics, clinical characteristics									
Gender, male n (%)	2,094 (62.0)	1,623 (63.3)	471 (57.8)	0.005	0.114	464 (59.2)	456 (58.2)	0.72	0.021
Age (yr) median (IQR)	66.5 [55.7, 77.6]	66.8 [56.6, 77.2]	65.2 [53.5, 78.9]	0.209	0.066	64.4 [55.0, 75.3]	65.5 [53.6, 79.0]	0.17	0.059
Weight (kg) median (IQR)	80.1 [68.0, 94.4]	80.3 [68.0, 93.9]	80.0 [67.9, 95.6]	0.762	0.042	82.0 [68.5, 95.5]	80.0 [67.9, 95.4]	0.465	0.011
Ethnicity, white (n %)	2,360 (69.9)	1,807 (70.5)	553 (67.9)	0.159	0.058	518 (66.1)	529 (67.5)	0.592	0.03
Chronic pulmonary disease n (%)	754 (22.3)	547 (21.4)	207 (25.4)	0.018	0.096	193 (24.6)	195 (24.9)	0.953	0.006
Chronic heart disease n (%)	811 (24.0)	607 (23.7)	204 (25.0)	0.464	0.031	196 (25.0)	196 (25.0)	1	<0.001
Diabetes, n (%)	933 (27.6)	680 (26.5)	253 (31.0)	0.014	0.1	228 (29.1)	243 (31.0)	0.441	0.042
Hypertension, n (%)	2,067 (61.2)	1,619 (63.2)	448 (55.0)	<0.001	0.168	419 (53.4)	444 (56.6)	0.223	0.064
Heart rate (bpm)	86.0 [78.0, 101.0]	84.0 [77.0, 97.0]	94.0 [80.0, 110.0]	<0.001	0.387	87.0 [77.0, 102.2]	94.0 [80.0, 110.0]	<0.001	0.227
MAP (mmHg)	77.0 [68.0, 88.0]	78.0 [68.0, 87.0]	76.0 [66.0, 89.0]	0.135	0.025	78.0 [68.0, 87.0]	76.0 [66.0, 89.0]	0.324	0.009
Respiratory rate (bpm)	17.0 [14.0, 22.0]	16.0 [14.0, 20.0]	20.0 [16.0, 24.0]	<0.001	0.441	18.0 [15.0, 22.0]	20.0 [16.0, 24.0]	<0.001	0.241
SpO2 (%)	99.0 [96.0, 100.0]	100.0 [97.0, 100.0]	98.0 [95.0, 100.0]	<0.001	0.327	99.0 [96.0, 100.0]	98.0 [95.0, 100.0]	<0.001	0.152
Temperature (°C)	36.6 [36.2, 37.1]	36.6 [36.1, 37.0]	36.8 [36.4, 37.3]	<0.001	0.315	36.6 [36.2, 37.1]	36.8 [36.4, 37.3]	<0.001	0.245
INR (IQR)	1.5 [1.3, 1.8]	1.5 [1.4, 1.8]	1.5 [1.3, 1.7]	<0.001	0.242	1.5 [1.3, 1.7]	1.5 [1.3, 1.7]	0.372	0.019
APTT(s) (IQR)	33.1 [28.9, 39.7]	32.9 [28.9, 39.8]	33.6 [29.2, 39.4]	0.169	0.011	32.5 [28.6, 39.2]	33.5 [29.3, 39.4]	0.102	0.038
Hemoglobin (g/L) (IQR)	10.0 [8.6, 11.8]	9.8 [8.4, 11.4]	10.9 [9.4, 12.7]	<0.001	0.449	10.8 [9.1, 12.7]	10.8 [9.3, 12.7]	0.902	0.002
Minimum platelet (10³/μl) (IQR)	137.0 [100.0, 192.0]	133.0 [97.0, 181.0]	157.0 [107.0, 233.0]	<0.001	0.386	153.0 [105.0, 220.2]	156.0 [105.0, 223.2]	0.305	0.056
WBC (10³/μl) (IQR)	10.9 [7.3, 15.8]	10.8 [7.4, 15.4]	11.3 [6.8, 17.0]	0.38	0.127	10.9 [7.3, 16.4]	11.1 [6.8, 16.9]	0.578	0.025
Ventilation, n (%)	1,610 (47.7)	1,277 (49.8)	333 (40.9)	<0.001	0.181	300 (38.3)	323 (41.2)	0.256	0.06
Vasopressor, n (%)	2,118 (62.7)	1,687 (65.8)	431 (52.9)	<0.001	0.266	412 (52.6)	418 (53.3)	0.8	0.015
SIC score, n (%)				<0.001	0.221			0.917	0.021
4	1,848 (54.7)	1,335 (52.1)	513 (62.9)			479 (61.1)	484 (61.7)		
5	893 (26.4)	713 (27.8)	180 (22.1)			177 (22.6)	178 (22.7)		
6	636 (18.8)	514 (20.1)	122 (15.0)			128 (16.3)	122 (15.6)		
SOFA score	7.0 [5.0, 9.0]	6.0 [5.0, 9.0]	8.0 [5.0, 10.0]	<0.001	0.217	7.0 [5.0, 10.0]	8.0 [5.0, 10.0]	0.198	0.02
SAPS II score median (IQR)	37.0 [29.0, 47.0]	36.0 [29.0, 46.0]	40.0 [31.0, 50.0]	<0.001	0.193	38.0 [30.0, 49.0]	40.0 [31.0, 50.0]	0.234	0.031
Hospital.stays (d) median (IQR)	7.0 [4.9, 11.5]	6.5 [4.8, 10.7]	8.5 [5.4, 14.3]	<0.001	0.239	7.1 [4.7, 12.3]	8.5 [5.4, 14.4]	<0.001	0.128
ICU stays (d) median (IQR)	2.3 [1.4, 4.0]	2.2 [1.3, 3.5]	3.0 [1.9, 5.4]	<0.001	0.355	2.4 [1.5, 4.0]	3.0 [1.9, 5.5]	<0.001	0.271

Abbreviations: IQR, interquartile range; MAP, mean arterial pressure; SPO2, oxygen saturation; WBC, white blood cell; INR, international normalized ratio; PT, prothrombin time; APTT, activated partial thromboplastin time; SIC, sepsis-induced coagulopathy; SOFA, sequential organ failure assessment; SAPS II, simplified acute physiology score II; ICU, intensive care unit.

## Outcomes

### PSM analysis on primary and secondary outcomes

After PSM, 1,568 patients were enrolled, 784 cases receiving UHF treatment were matched with 784 cases that did not receive UHF except for heart rate, respiratory rate, temperature, hospital and ICU stays, and the SMDs of other variables were <0.1, indicating that the baseline variables in the two groups had similar distributions (Table 1). The prematched mortality rates were higher in patients with heparin use than in those without heparin use (ICU, 11.7% vs. 11.2%, hazard ratio (HR) 0.54, 95% confidence interval (CI) [0.41–0.70] *p* < 0.001, hospital mortality, 15.5% vs. 13.8%, HR 0.73, 95% CI [0.58–0.91], *p* = 0.005). However, after PSM, there was significant effect on primary and secondary outcomes with UFH after PSM (ICU mortality, hazard ratio [HR] 0.69, 95% confidence interval [CI] 0.52–0.92; 7-day, HR 0.70, 95% CI 0.49–0.99; 14-day, HR 0.68, 95% CI 0.50–0.92; 28-day, HR 0.72, 95% CI 0.54–0.96; hospital mortality, HR 0.74, 95% CI 0.57–0.96) (Table 2).

**TABLE 2:** Association between heparin use and clinic outcomes in patients with SIC.

	Propensity score matching							
	Before				After			
Pre-matched cohort	Control group	UFH group	HR (95% CI)	*p*-value	Control group (n = 784)	UFH group (n = 784)	HR (95% CI)	*p*-value
	(n = 2,562)	(n = 815)						
Primary outcome								
ICU mortality n (%)^a^	286 (11.2)	95 (11.7)	0.54 (0.41, 0.70)	<0.001	119 (15.2)	91 (11.6)	0.69 (0.52, 0.92)	0.011
Secondary outcomes								
7-day mortality n (%)^b^	257 (10.0)	86 (10.6)	0.76 (0.55, 1.04)	0.085	106 (13.5)	80 (10.2)	0.70 (0.49, 0.99)	0.043
14-day mortality n (%)^b^	343 (13.4)	119 (14.6)	0.80 (0.61, 1.06)	0.128	140 (17.9)	112 (14.3)	0.68 (0.50, 0.92)	0.011
28-day mortality n (%)^b^	498 (19.4)	201 (24.7)	0.99 (0.77, 1.28)	0.959	164 (24.9)	151 (19.3)	0.72 (0.54, 0.96)	0.024
Hospital.mortality n (%)^a^	353 (13.8)	126 (15.5)	0.73 (0.58, 0.91)	0.005	148 (18.9)	120 (15.3)	0.74 (0.57, 0.96)	0.024

^a^ adjust for age, gender, ethnicity, weight, heart rate, MAP, respiratory rate, SpO2, WBC, temperature, hemoglobin, platelet, INR, APTT, ventilation, vasopressor, SAPS II, hypertension, diabetes and chronic heart disease.

^b^ adjust for ethnicity, weight, heart rate, respiratory rate, SpO2, WBC, temperature, hemoglobin, platelet, INR, APTT, ventilation, vasopressor, SAPS II, hypertension; CI, confidence interval.

### Marginal structural cox model and stratification analysis for ICU mortality

**FIGURE 2:**
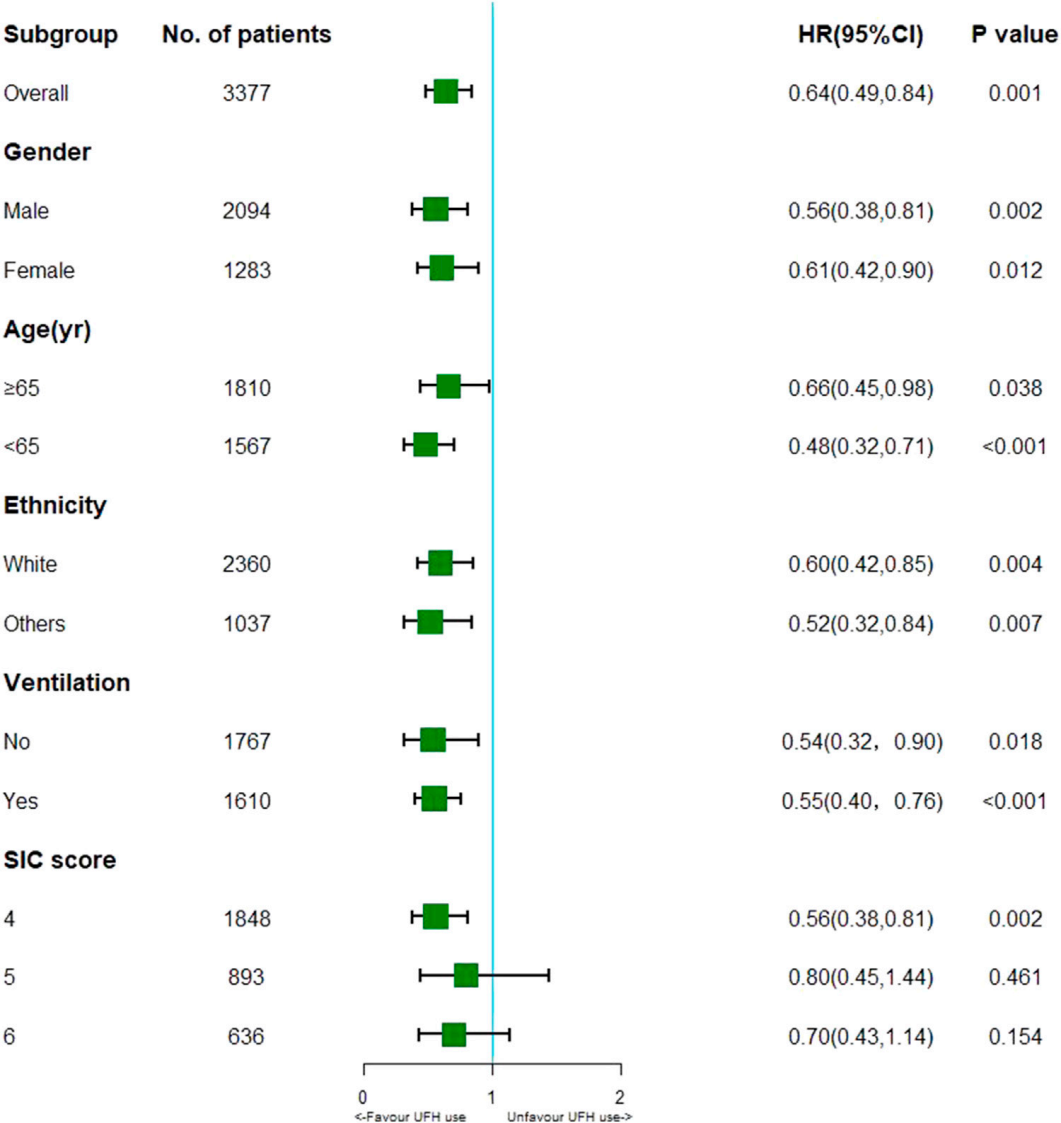
Results of ICU mortality in overall population with marginal structural Cox model and stratification analysis.

To assess the effectiveness of UFH on ICU mortality, we performed MSCM analysis on UFH according to time-varying confounding APTT for UHF. As demonstrated by MSCM analysis, UFH administration decreased ICU mortality (HR, 0.64; 95% CI, 0.49–0.84; *p* = 0.001) among general patients with SIC. Stratification analysis revealed that the administration of heparin decreased the risk of ICU mortality among patients with SIC scores of 4 (HR, 0.56; 95% CI, 0.38–0.81; *p* = 0.002). Cases with SIC scores of 5 or 6 exhibited distinct effects, and UFH treatment had no significant impact on ICU mortality. (Figure 2). In patients with SIC, 6,250–13,750 IU of mortality heparin decreased ICU mortality relative to the control group (Table 3).

**TABLE 3:** Dose-response relationship between heparin and ICU mortality in patients with SIC.

Daily UFH usage (control group as reference)	No. of patients^a^	HR (95% CI)	*p*-value
3,750–6250 IU	170	0.78 (0.41, 1.45)	0.424
6,250–8750 IU	180	0.36 (0.18, 0.73)	0.004
8,750–11250 IU	231	0.49 (0.29, 0.83)	0.007
11,250–13750 IU	167	0.47 (0.25, 0.88)	0.018
>13,750 IU	67	0.47 (0.13, 1.61)	0.227

^a^ The number of patients with UFH, administration.

### Subgroup analysis on primary and secondary outcomes in patients with SIC scores of 4

In the subgroup analysis, UFH was found to be beneficial for septic patients with SIC scores of 4, resulting in improved survival outcomes, including ICU mortality (HR: 0.51; 95% CI 0.34–0.76; *p* = 0.001), but 7-day mortality (HR: 0.75; 95% CI 0.47–1.22; *p* = 0.250), and 14-day mortality (HR: 0.78; 95% CI 0.50–1.22; *p* = 0.280), 28-day mortality (HR: 1.08, 95% CI 0.74–1.58, *p* = 0.697) and hospital mortality (HR, 0.73; 95% CI 0.52–1.04, *p* = 0.080) were not reduced (Table 4).

**TABLE 4:** Association of heparin use and mortality outcome in the patients with SIC score 4 with propensity score analysis.

SIC score 4 (n = 963)	Control group (n = 479)	UFH group (n = 484)
			HR (95% CI)	*p*-value
Primary outcome				
ICU mortality, n (%)^a^	56 (11.7)	51 (10.5)	0.51 (0.34, 0.76)	0.001
Secondary outcomes				
7-day mortality, n (%)^b^	51 (10.7)	44 (9.1)	0.75 (0.47, 1.22)	0.250
14-day mortality, n (%)^b^	64 (13.4)	59 (12.2)	0.78 (0.50, 1.22)	0.280
28-day mortality, n (%)^b^	76 (15.9)	86 (17.8)	1.08 (0.74, 1.58)	0.697
Hospital mortality, n (%)^a^	66 (13.8)	65 (13.4)	0.73 (0.52, 1.04)	0.080

^a^ adjust for ethnicity, temperature, WBC, INR, APTT, and vasopressor.

^b^ adjust for ethnicity, temperature, respiratory rate, SpO_2_, WBC, INR, SAPS II, vasopressor and ventilation.

### Sensitivity analysis

The significant known and measured risk factors for ICU mortality after PSM within the Cox proportional hazards model included ethnicity (HR, 1.69, 95% CI, 1.15–2.49), temperature (HR, 0.66, 95% CI, 0.58–0.75), INR (HR, 1.47, 95% CI, 1.13–1.91), APTT (HR, 1.01, 95% CI, 1.00–1.02), white blood cell (HR, 1.04, 95% CI, 1.02–1.05), SAPS II score (HR, 1.04, 95% CI, 1.03–1.05), vasopressor (HR, 2.30, 95% CI, 1.48–3.57) and chronic heart disease (HR, 1.58, 95% CI, 1.06–2.34) (Supplementary Table S3).

We performed an E-value analysis to assess the sensitivity to unmeasured confounding (https://www.evalue-calculator.com/evalue/). The primary findings were robust, unless there were unmeasured confounders, a low relative risk of ICU mortality, and an HR higher than 3.33 (upper limit 6.80), meaning that residual confounding could explain the observed association if there was an unmeasured covariate having a relative risk association >3.33 with both ICU mortality and heparin administration. Thus, it was unlikely that an unmeasured or unknown confounder would have a substantially greater impact on ICU mortality (relative risk exceeding 3.33) than these known risk factors.

## Discussion

Early administration of unfractionated heparin reduces 30-day mortality, whereas LMWH at therapeutic doses reduces mortality in COVID-19 patients (Lawler et al., 2021; Rentsch et al., 2021; Spyropoulos et al., 2021). According to the most recent data on inpatients, serum chemokine and cytokine levels increased in patients with severe COVID-19, similar to that in patients with sepsis. However, sepsis is a highly heterogeneous syndrome, and additional research is required to determine the timing, dosage, and efficacy of unfractionated heparin in the management of septic complications. Unfractionated heparin administration to patients with a SIC score of 4 was associated with improved survival parameters, including ICU mortality, but not with reduced 7-day, 14-day, 28-day mortality or hospital mortality as suggested by the MIMIC-IV data. Stratification and subgroup analyses revealed that patients with SIC given 6,250–13750 IU/day unfractionated heparin had a decreased risk of ICU mortality.

In addition to its anticoagulant properties, unfractionated heparin exhibits anti-inflammatory, anticomplement, immune modulation, and antihistone effects, according to certain clinical studies and animal research (Wildhagen et al., 2014; Li and Ma, 2017; Peng et al., 2021; Tang et al., 2021). The nonanticoagulant heparin has been implicated as an effective anti-histone drug for histone infusion models (Peng et al., 2021) and has the potential to attenuate multiple organ dysfunction while improving patient survival. A report described the novel *in vitro* immunomodulation mechanism of heparin; according to the findings, nonanticoagulant heparin administered to a septic mouse model may protect against sepsis by inhibiting the circulation of histones (Cheng et al., 2019). Our data demonstrated that in patients with SIC, administration of 6,250–13750 IU heparin reduced ICU mortality, which may be due to the nonanticoagulant effect of the underlying mechanisms. As suggested by a recent report, heparin suppressed the caspase-11-mediated immunity and mortality in sepsis, independent of its anticoagulant effect (Yang et al., 2019). Heparin inhibited the interactions between high-mobility group box-1 protein (HMGB1) and LPS, preventing the degradation of macrophage glycocalyx by heparanase. The aforementioned events inhibit cytosolic LPS delivery within macrophages and caspase-11 activation (the cytosolic receptor of LPS that mediates sepsis-related mortality), thereby reducing organ damage and increasing the survival rate (Tang et al., 2021). On the basis of the above findings, heparin may be an effective treatment for sepsis; however, it is urgently necessary to investigate its mechanism, timing, and dosage.

According to MSCM, heparin administration reduced ICU mortality among SIC patients in this study (HR 0.64). Based on subgroup analyses, heparin administration decreased in-hospital mortality among SIC cases (HR, 0.74) (Zou et al., 2022). The effect sizes of heparin were comparable to those in our previous study. Nonetheless, the previous study did not conduct a subgroup analysis of SIC with MSCM, which may have introduced bias. We conducted a study on SIC as a result. Patients with a SIC score of 4 had decreased ICU mortality. This differs from a previous study of the *post hoc* subgroup analyses on the whole-nation multicenter retrospective registry in Japan, which demonstrated that anticoagulation treatment exhibited possible survival benefits only among SIC cases in the high-risk subset (SOFA score 13–17) and not in low-to-moderate risk septic cases (Yamakawa et al., 2016). Another retrospective study in septic patients with SIC observed that unfractionated heparin (UFH) administration decreased 28-day (HR, 0.323; 95% CI, 0.258–0.406; *p* < 0.001) as well as in-hospital (HR, 0.380; 95% CI, 0.307–0.472; *p* < 0.001) mortality, with no increase in gastrointestinal bleeding or intracranial hemorrhage (Peng et al., 2021). However, another study reported no marked effect on 28-day mortality (Jaimes et al., 2009). Due to the absence of time-varying confounding factors in the aforementioned study, the efficacy of heparin in the treatment of SIC remains uncertain. In the current study, heparin had significant effect on ICU mortality before or after PSM. At the same time, stratification analysis revealed that heparin treatment reduced ICU mortality risk in cases with a SIC score of 4. What is the underlying explanation for this result? To account for time-varying confounding, a previous study may have used only Cox regression analysis stratified by propensity scores rather than MSCM. MSCM was utilized to analyze both fundamental and time-dependent confounding variables, which was a strength Clinical use of heparin varies with time, as determined by previous APTT levels, and heparin affects subsequent APTT. Thus, complex and dynamic relationships may exist between the use of heparin, APTT and mortality. Dupuis C et al evaluated the effect of red blood cell (RBC) transfusion on sepsis-related mortality using MSCM analysis. Their clinical situation was similar to ours in that RBC transfusion was measured based on previous hemoglobin levels and could affect subsequent hemoglobin levels (Dupuis et al., 2017). MSCM is typically also utilized in other situations involving time-varying interventions (de Keyser et al., 2014; Karim et al., 2014). We used risk factor analysis with E-value analysis and the multivariate Cox proportional hazard model to assess data regarding unmeasured confounding variables. The results suggested that the unmeasured confounder may not have had a significant impact on ICU mortality compared to the known risk factors.

Notably, our results must be interpreted in light of our work’s limitations. This study was conducted following an EHR, with data collected through clinical practice Therefore, cohort screening may not be identical to the guidelines-based definition of sepsis. In spite of this, cases of sepsis as defined by the third definition of sepsis (infection plus acute alteration of the total. SOFA score ≥2 points) were identified. Second, due to the retrospective nature of the study, indication-related confounding could occur; therefore, PSM and MSCM were used to balance out the critical confounders. Thirdly, some patient variables were not collected according to the database, which may have led to bias or confounding. A sensitivity analysis of the E-value was conducted to quantify potential indications of these non-extracted confounders. According to our findings, the non-extracted confounding variable may not influence therapeutic efficacy. Fourth, multiple subgroup analyses were conducted, which could have led to false-positive findings. Both PSM and MSCM analyses produced identical outcomes, validating the dependability of our findings.

## Conclusion

Unfractionated heparin administration appears to be associated with improved survival outcomes, including ICU mortality, 7-day, 14-day, 28-day mortality and hospital mortality. In addition, patients with a SIC who received 6,250–13750 IU unfractionated heparin per day had a lower ICU mortality rate than those who did not receive heparin. Subgroup analysis further demonstrates heparin therapy decreased ICU mortality primarily in patients only with SIC score of 4.

## Data Availability

The datasets presented in this study can be found in online repositories. The names of the repository/repositories and accession number(s) can be found below: These data are available at https://mimic-iv.mit.edu/.
