# Supplementary material for: Effectiveness of early heparin therapy on outcomes in critically ill patients with sepsis-induced coagulopathy
Source: Front Pharmacol. 2023 May 15;14:1173893. doi: 10.3389/fphar.2023.1173893 (PMC10225678; doi:10.3389/fphar.2023.1173893)
Supplement: Supplementary file 1 [file Table1.docx]

***Supplementary materials***

**Table S1 diagnosis criteria of sepsis-induced coagulopathy**

|  | **points** | **items** |
| --- | --- | --- |
| Platelet count (10^9^/L) | 2 | < 100 |
|  | 1 | ≥ 100, <150 |
| INR | 2 | > 1.4 |
|  | 1 | >1.2, ≤1.4 |
| Total SOFA score | 2 | ≥ 2 |
|  | 1 | 1 |

Abbreviations: INR, international normalized ratio.

Note: total SIC score is 4 or more with sum of SOFA score and coagulation criteria exceeding 2. Total SOFA score is the sum of four items(respiratory SOFA, cardiovascular SOFA, hepatic SOFA, and renal SOFA).

Table S2. Missing number (%) for included variables in the datasets

| **Variables** | **Missing (%)** |
| --- | --- |
| Age | 0 (0) |
| Gender | 0 (0) |
| Weight | 235(7.0) |
| WBC | 0 (0) |
| Hemoglobin | 1 (0) |
| Platelet | 0 (0) |
| INR | 12 (0.4) |
| APTT | 26 (0.8) |
| SpO_2_ | 0(0) |
| Temperature | 44 (1.3) |
| MAP | 4 (0) |
| Heart rate | 2 (0) |
| Respiratory rate | 15 (0.4) |
| Hypertension | 0 (0) |
| Diabetes | 0 (0) |
| Chronic heart disease | 0 (0) |
| Chronic pulmonary disease | 0 (0) |
| Vasopressor | 0 (0) |
| Mechanical ventilation | 0 (0) |
| SOFA | 0 (0) |
| SAPS II | 0 (0) |
| SIC score | 0 (0) |
| ICU mortality | 0 (0) |
| Hospital mortality | 0 (0) |
| Length of ICU stay | 0 (0) |
| Length of hospital stay | 0 (0) |

Abbreviations: WBC, white blood cell; INR, international normalized ratio; APTT, activated partial thromboplastin time; MAP, mean arterial pressure; SIC, sepsis-induced coagulopathy; SOFA, sequential organ failure assessment; SAPS II, simplified acute physiology score II .

Table S3. Cox regression model after propensity score matching in patients with SIC score 4

| **Variables** | **HR (95%CI)** |  | ***P value*** |
| --- | --- | --- | --- |
| **Gender** |  |  |  |
| Female | reference |  |  |
| Male | 1.13 (0.76,1.67) |  | 0.551 |
| Age | 1.01 (1.00,1.02) |  | 0.066 |
| **Ethnicity** |  |  |  |
| White | reference |  |  |
| Others | 1.69（1.15,2.49） |  | 0.008 |
| Weight | 0.99 (0.99,1.00) |  | 0.108 |
| Temperature | 0.66（0.58,0.75） |  | <0.001 |
| Heart rate | 1.00（0.99,1.00） |  | 0.374 |
| Respiratory rate | 1.03（1.00,1.05） |  | 0.076 |
| MAP | 1.00（0.99,1.01） |  | 0.819 |
| SPO_2_ | 0.98 (0.95,1.02) |  | 0.327 |
| INR | 1.47（1.13,1.91） |  | 0.004 |
| APTT | 1.01 (1.00,1.02) |  | 0.026 |
| WBC | 1.04 (1.02,1.05) |  | <0.001 |
| Hemoglobin | 1.06 (0.98,1.14) |  | 0.121 |
| SAPS II score | 1.04 (1.03,1.05) |  | <0.001 |
| **Ventilation** |  |  |  |
| No | reference |  |  |
| Yes | 1.48 (0.99,2.24) |  | 0.059 |
| **Vasopressor** |  |  |  |
| No | reference |  |  |
| Yes | 2.30(1.48,3.57) |  | <0.001 |
| **Chronic heart disease** |  |  |  |
| No | reference |  |  |
| Yes | 1.58 (1.06,2.34) |  | 0.023 |
| **Chronic pulmonary disease** |  |  |  |
| No | reference |  |  |
| Yes | 1.26 (0.84,1.90) |  | 0.266 |
| **Diabetes** |  |  |  |
| No | reference |  |  |
| Yes | 0.72(0.46,1.11) |  | 0.139 |
| **Hypertension** |  |  |  |
| No | reference |  |  |
| Yes | 0.70 (0.48,1.11) |  | 0.068 |

Abbreviations: MAP, mean arterial pressure; WBC, white blood cell; INR, international normalized ratio; APTT, activated partial thromboplastin time; SAPS II, simplified acute physiology score II.
